# The Pathogenesis, Potential Biomarkers and Novel Therapeutic Strategies for Tubulointerstitial Nephritis in Systemic Lupus Erythematosus—A Narrative Review

**DOI:** 10.3390/ijms262210903

**Published:** 2025-11-10

**Authors:** Chang-Youh Tsai, Tsai-Hung Wu, Shuo-Ming Ou, Hui-Ting Lee, Chieh-Yu Shen, Cheng-Hsun Lu, Wan-Hao Tsai, Chia-Li Yu

**Affiliations:** 1 Division of Immunology & Rheumatology, Department of Medicine, Fu-Jen Catholic University Hospital, College of Medicine, Fu Jen Catholic University, New Taipei City 24352, Taiwan; augustus81917@yahoo.com.tw; 2 Division of Nephrology, Department of Medicine, Taipei Veterans General Hospital, Faculty of Medicine, National Yang-Ming Chiao-Tung University, Yang-Ming Campus, Taipei 112304, Taiwan; thwu@vghtpe.gov.tw (T.-H.W.); okokyytt@gmail.com (S.-M.O.); 3 Division of Allergy, Immunology & Rheumatology, Department of Internal Medicine, Mackay Memorial Hospital, New Taipei City 25245, Taiwan; htlee1228@gmail.com; 4 Department of Medicine, Mackay Medical College, New Taipei City 25245, Taiwan; 5 Division of Rheumatology, Immunology & Allergy, Department of Internal Medicine, National Taiwan University Hospital, National Taiwan University College of Medicine, Taipei 100115, Taiwan; chiehyushen@ntu.edu.tw (C.-Y.S.); b98401085@ntu.edu.tw (C.-H.L.)

**Keywords:** systemic lupus erythematosus, lupus nephritis, tubulointerstitial nephritis, anti-vimentin autoantibodies, interleukins, interstitial fibrosis/tubular atrophy

## Abstract

Kidney diseases in patients with SLE include glomerulonephritis (GN), tubulointerstitial nephritis (TIN) and vasculitis alone or in combination. Immune complex (IC) deposition with complement activation in renal glomeruli causes lupus GN. However, IC deposition can also occur in the tubular basement membrane, renal interstitium, peritubular capillaries and arteries/arterioles to elicit inflammatory responses. TIN is usually associated with more severe GN with inflammation induced by IC. Immunopathologically, the aberrant presentation of T cell subpopulations, T_h1_, T_h2_, T_h9_, T_h17_, Treg and follicular T helper cells (T_fh_), is closely implicated in TIN in SLE. In addition, M_1_/M_2_ macrophages and more specific dendritic cells (DCs) contribute to the inflammatory reactions of SLE-TIN. TIN may also present alone (isolated TIN) in apparently normal glomeruli or class I GN. It is intriguing that lupus nephritis constitutes two different pathological predilections, i.e., GN and tubulointerstitial inflammation. Alternatively, these two types may represent a continuous spectrum of inflammatory renal damages. In the present review, we will discuss in detail the pathology/immunopathogenesis, likely specific biomarkers/predictors and novel therapeutic designs for SLE-tubulointerstitial inflammation. In addition, we also raise several plausible investigation methods in SLE-tubulointerstitial inflammation that may help further elucidate this setting of perplexing renal diseases with rheumatic characteristics.

## 1. Introduction

Systemic lupus erythematosus (SLE) is an archetype of systemic autoimmune disease associated with immune-mediated organ damage. A total of 40-50% of SLE patients develop lupus nephritis (LN) and 10% of LN patients develop end-stage renal disease (ESRD) after one decade of illness [1]. Renal diseases in SLE are variably implicating glomerular, tubulointerstitial (TI) and/or vascular compartments whereas none of them are mutually exclusive [2]. These different pathological findings are mediated by immune complex (IC) deposition in tissues of the renal cortex and medulla including glomeruli, tubulointerstitium and vasculature alone or in combination. IC deposition in renal glomeruli causes lupus glomerulonephritis (GN). Extraglomerular IC depositions can be found in the tubular basement membrane (TBM), interstitium, peritubular capillaries, and arteries/arterioles that are variably demonstrated in up to 40% LN [3,4,5,6]. The GN is mediated mainly by autoantibodies or preformed IC deposition with complement activation, whereas the pathology and pathogenetic factors for tubulointerstitial nephritis (TIN) are more intricate, including IC deposition, innate inflammatory process, cytotoxic T cell response and inflammation intermingled with atrophy mediated by kidney resident cells. Clinical observations unveiled that TIN is usually associated with severe GN such as those seen in class IV LN. But isolated TIN originating from a unique Ig class can be found in GN caused by variable Ig classes in SLE [7,8]. Hayashi et al. demonstrated that differences in the IgG subclass depositions may reflect differences in the formation and deposition of glomerular or extraglomerular ICs [9]. Their results imply that the antibody composition of glomerular and extraglomerular IC depositions may differ. Moreover, Leatherwood et al. demonstrated in biopsies that renal outcome may be determined by interstitial fibrosis, tubular atrophy and vascular injury [10].

**Figure 1:**
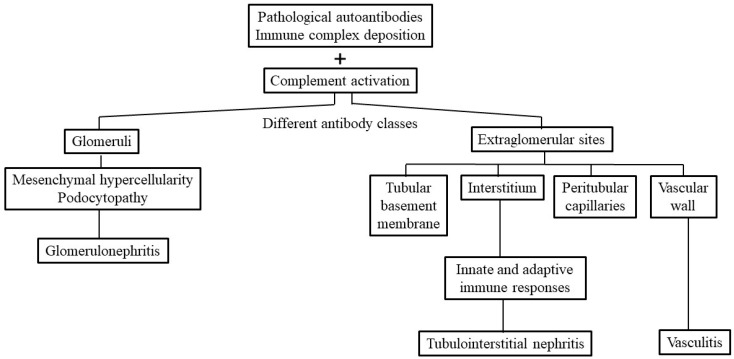
Different pathological autoantibodies and the preformed antigen–antibody immune complexes (ICs) may deposit in the glomerular and/or extraglomerular sites to induce tissue inflammation in patients with SLE. These extraglomerular sites may include tubular basement membrane, interstitium, peritubular capillaries and vascular walls. The complement activation can attract inflammatory cell infiltration and inflammation that may cause mesangial hypercellularity, podocytopathy and glomerulonephritis. On the other hand, the ICs deposited in the interstitium may elicit innate and adaptive immune responses resulting in tubulointerstitial inflammation. Once the IC deposition occurs in the vascular wall, a vasculitis develops.

In addition to autoantibodies, IC deposition and complement activation-mediated tissue inflammation/destruction, kidney resident cells, including podocytes and tubular epithelial cells, may also be involved in lupus renal damage. Podocytes are highly differentiated epithelial cells within the Bowman’s capsule, attaching to the glomerular basement membrane (GBM) via cytoskeletal anchors, namely, α3β1-integrins. These specified cells work together with fenestrated endothelial cells on opposing sides of GBM in formulating the final glomerular filtrating barriers (slit diaphragm) [11,12]. This structure is made up of a special tight junction between foot processes of neighboring podocytes. Thus, proteinuria in LN is associated with the magnitude of foot process/effacement loss rather than mesangial IC deposition or hypercellularity. Han et al. revealed that LN proteinuria is associated with podocytopathy rather than IC formation/deposition in glomeruli [13]. The crucial roles of podocytes in LN have been extensively reviewed by Maeda et al. and Koga et al. [12,14]. A brief outline of LN classification correlated to IC deposition is depicted in Figure 1.

In this report, we shall mainly raise a scheme to account for why TIN can be induced by the interactions among autoantibodies, innate and adaptive immune cells and kidney resident cells, eventually leading to renal tubular atrophy/fibrosis.

## 2. Pathologic Findings and Immunopathogenic Mechanisms in SLE-TIN

### 2.1. Immunopathological Findings in SLE-Tubulointerstitial Inflammation

Many authors have revealed that changes in naïve/memory B cells and plasma cells are present in LN, causing the production of diverse autoantibodies against dsDNA, nucleosome, Ro, Smith antigen, C1q, α-actinin, annexin II and ribosomal P protein, leading to resident renal cell damage [15,16]. Recently, Yap and Lai showed that aberrantly presenting T lymphocytes, especially T-helper subpopulations, including T_h1_, T_h2_, T_h9_, T_h17_, Treg and follicular T helper cells (T_fh_), are also involved in SLE and LN [17].

**Figure 2:**
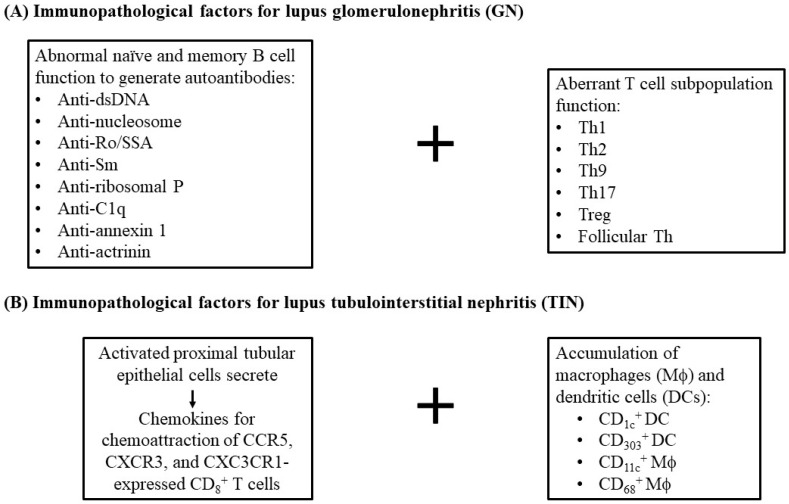
Different immunopathological factors participating in the pathogenesis of lupus glomerulonephritis (GN) and tubulointerstitial nephritis (TIN). (**A**) Different kinds of autoantibodies, including anti-dsDNA, anti-Sm, anti-Ro, anti-nucleosome, anti-ribosomal P and anti-connective tissue components, are involved in GN. In addition, different helper T cell subpopulations such as Th1, Th2, Th9, Th17, Treg and follicular Th are formed in the site of GN. However, as shown in (**B**), the kidney resident proximal tubular epithelial cells can secrete chemokines for chemo-attracting CD8+ T cell population after activation. Furthermore, macrophages and dendritic cells accumulate in the tubulointerstitial tissue to induce TI inflammation.

Furthermore, activated proximal tubular epithelial cells could release chemokines to attract T cells expressing CCR5, CXCR3, CX3CR1 and CD1c^+^ myeloid dendritic cells (mDC) expressing fractalkine-CX3CR1 [18,19]. Cheng et al. investigated dendritic cells (DCs) in renal biopsies from patients with minimal change disease, LN and acute TIN (ATIN) [20]. They found CD1c^+^ DCs and CD303^+^ DCs were present in ATIN. These data have suggested the important roles of renal tubular epithelial cells (RTECs) in immune pathophysiology of TIN and SLE-TIN [21]. Instead, CD11c^+^/CD68^+^ macrophages are also involved in LN [22,23,24]. Zhang et al. [25] reported that CD8^+^ T cell infiltration is correlated to tubulointerstitial inflammation [23], interstitial fibrosis and tubular atrophy. Additionally, tubulointerstitial inflammation with CD8^+^ T cells > 130/mm^2^ was associated with ESRD progression [25]. Azoicӑi et al. identified that CD8^+^ T lymphocyte count was higher than CD4^+^ T count in intra-/peri-glomeruli and interstitia in biopsied specimens from LN patients, which correlated closely to tubulointerstitial inflammation [26]. The evidence strongly supports the major role of CD8^+^ rather than CD4^+^ T lymphocytes in tubulointerstitial inflammation. Possible factors participating in the development of immunopathological abnormalities of LN and SLE-tubulointerstitial inflammation are summarized in Figure 2.

### 2.2. Immunopathogenic Basis for SLE-TIN

#### 2.2.1. Role of Anti-dsDNA Antibodies

Evidently, dsDNA-anti-dsDNA IgG IC deposition in renal glomeruli elicits lupus GN. These ICs may also deposit in renal tubules to induce inflammation and fibrosis in the tubulointerstitium [27]. Human polyclonal anti-dsDNA antibodies can directly bind to human proximal RTECs, inducing tubulointerstitial inflammation and fibrosis in LN. Yung et al. found sequentially increased production of TNF-α, IL-1β and IL-6 in incubating human RTEC with anti-dsDNA [28]. These results suggest that renal resident cells (podocytes, mesangial cells and epithelial cells) are involved in LN pathogenesis [29]. In addition, Chen et al. noted that serum IgE anti-dsDNA autoantibodies are prevalent, being associated with tubulointerstitial inflammation in proliferative LN [30].

#### 2.2.2. Role of Anti-Monomeric or Anti-Modified CRP (Anti-mCRP) Antibodies in SLE-Tubulointerstitial Inflammation

Plasma C-reactive protein (CRP) is produced by hepatocytes and exists in at least two conformational forms: native pentameric CRP and monomeric/modified CRP (mCRP). The normal CRP is predominantly produced by hepatocytes, whereas mCRP is generated partly by RTECs, neurons, lymphocytes or alveolar macrophages [31,32,33,34]. CRP belongs to the pentraxin family. It engages [Ca^++^]-dependent ligand-binding plasma protein to process pro- and/or anti-inflammatory activities. However, under certain circumstances such as altered pH, high urea, or low calcium concentration, native CRP may dissociate irreversibly into monomers or distinct antigenic isomers with unique physiochemical characteristics [35,36].

Zhao’s group demonstrated autoantibodies against mCRP in the sera of LN patients that are associated with disease activity and TIN [37,38,39]. The key epitope of mCRP exists in amino acid 35–37 [39]. Furthermore, they unveiled that the anti-mCRP_199–206_ antibodies can aggravate tubulointerstitial inflammation in LN. These results suggest that circulatory antibodies against mCRP_199–206_ can become a biomarker for TI lesion and a potential therapeutic target in LN [40]. Moreover, the authors also demonstrated that urinary mCRP is closely associated with TI lesions in LN [41].

#### 2.2.3. Other Autoantibodies That Induce Human Lupus TIN

It is believed tubulointerstitial inflammation rather than GN severity can predict progression of renal failure [42,43]. Severe tubulointerstitial inflammation is commonly associated with enhanced in situ adaptive immunity characterized by tertiary lymphoid aggregation [4]. Among the targets of adaptive immunity, vimentin, a filamentous antigen, is recognized by *in situ* activated B cells. Kinloch et al. found that vimentin is highly expressed by tubulointerstitial inflammatory cells and “anti-tubulointerstitial inflammation” antibodies preferentially bind to the inflamed interstitium [44]. Moreover, high-titer IgG anti-vimentin antibodies (AVAs) are linked to severe tubulointerstitial inflammation. They later demonstrated that AVAs are unique compared to other common autoantibodies such as anti-dsDNA in distribution and poor response to conventional therapies [45]. However, the somatic hypermutation of IgG AVA leads to both increased vimentin affinity and poly-reactivity in *in vitro* enzyme-linked immunoassays [46].

#### 2.2.4. Viral Infection as a Possible Etiology of Tubulointerstitial Nephritis in SLE

Parvovirus B19 (PVB19) is a non-enveloped single-stranded DNA virus that infects only susceptible humans [47]. The infection is often subclinical but can occasionally become severe in certain immune-deranged conditions. Its implication in GN has been well documented, which is characterized by an immune complex deposition pattern mimicking autoimmune diseases, especially in the form of membranoproliferative glomerulonephritis [48,49]. It is possible that a PVB19-derived glomerular pathogen cross-reacts with nephritis-associated plasmin receptor to form autoantibodies against glomerular tissue [49]. On the other hand, PVB19 has been attributed to SLE with various end-organ manifestations [50]. The capsid antigen of PVB19 has been detected in podocytes, parietal epithelial cells and tubular epithelial cells in patients with nephritis that was associated with PVB19 infection [51,52]. Likewise, based on a similar mechanism, PVB19 may also play a role in TIN either in non-immune-mediated diseases such as hereditary spherocytosis [52] or in SLE.

## 3. Factors Directly Associated with Interstitial Fibrosis/Tubular Atrophy (IFTA) in LN-Tubulointerstitial Inflammation

**Figure 3:**
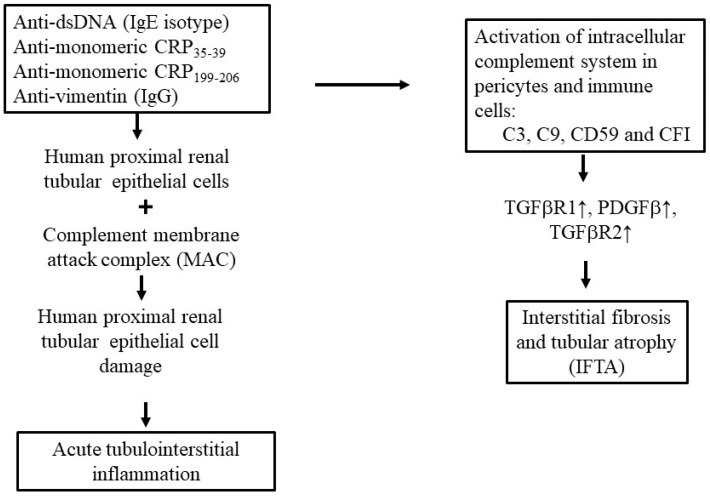
The roles of specific autoantibodies and the activated extracellular/intracellular complement system in kidney pericytes and immune-related cells in the development of acute tubulointerstitial inflammation and IFTA. Some particular autoantibodies such as IgE isotype anti-dsDNA, anti-monomeric CRP or IgG anti-vimentin not only activate serum complement system to induce MAC but also destroy the proximal renal tubular epithelial cells (RTECs). This may lead to acute tubulointerstitial inflammation. Alternatively, these particular autoantibodies may also activate the intracellular complement system in pericytes and immune cells. These intracellular complements including C3, C9, CD59 and CFI, can stimulate production of profibrotic TGFβR1, TGFβR2 and PDGFβ for IFTA. C: complement component; CD: cluster of differentiation; TGF: transforming growth factor; PDGF: plate-derived growth factor; CFI: complement factor 1 or C3b/C4b inactivator; IFTA: interstitial fibrosis/tubular atrophy. CRP: C-reactive protein; Ig: immunoglobulin; MAC: membrane attacking complex of the complement system. The black arrow indicates “stimulate”, “induce’, “evolve to”; small vertical arrow with arrow head on the top represents enhancement.

Low complements C3 and C4 are not clinically associated with IFTA. However, Wang et al. demonstrated thatdeposition of membrane attack complex (MAC) against tubule is associated with high-degree IFTA and proteinuria. These can be regarded as predictors for progression to ESRD [53]. Prior to these findings, Xavier group reported intracellular complement activations in pericytes and immune cells contributed to kidney fibrosis [54,55,56]. Wang et al. found that C9, CD59 (MAC inhibitor), C3 and factor I (complement factor I [CFI] or C3b/C4b inactivator) correlated with transforming growth factor beta receptor 1 (TGFβR1), platelet-derived growth factor beta (PDGFβ) and platelet-derived growth factor receptor beta (PDGFRβ), whereas C9, CD59 and C3 correlated with TFGβR2 by urine proteomics [57]. These results provided evidence to support that CFI, C3 and C9/CD59 ratio could become potential predictors for tubulointerstitial fibrosis in LN. Histologically, interstitial fibrosis in LN is characterized by infiltration of immune/inflammatory cells regarded as nonspecific “sear reaction” or inflammatory fibrosis (i-IFTA). I-IFTA is dominantly characterized by macrophage/T cell infiltrations. Malvica et al. reported that i-IFTA magnitude could be a predictor for glomerular filtration rate (GFR) loss, implicated as a prognostic indicator for end-stage LN [58]. Figure 3 summarizes various factors that contribute to the development of tubulointerstitial inflammation and IFTA in LN.

## 4. Specific or Nonspecific Biomarkers for SLE-TIN

Identification of specific biomarkers in SLE-TIN is quite important since TI injury in LN is associated with poor renal outcome [59].

### 4.1. Pentraxin 3 (PTX3) Is Closely Associated with TI Injury

Pentraxin is a family of acute-phase proteins featured by a cyclic multimeric structure serving as inflammatory biomarker and regulator of innate immunity to modulate complement activation, apoptotic cell clearance and maintenance of immune tolerance [60,61]. Pentraxin 3 (PTX3) is a prototypical long PTX formed by a C-terminal domain homologous to classic short pentraxins bonded with unrelated N-terminal 178 amino acid domain [62], which is rapidly produced at inflammatory sites by dendritic cells, macrophages, fibroblasts, endothelial cells and native renal cells [63,64,65]. It is different from short pentraxin CRP, which is strictly produced by hepatocytes. Pang et al. demonstrated significant increased circulatory and urinary PTX3 in SLE-tubulointerstitial inflammation that are also closely associated with urinary kidney injury molecule 1 (KIM-1), neutrophil gelatinase-associated lipocalin (NGAL), microalbumin and transferrin excretion, and pathologic scores for inflamed kidney in active LN [66].

### 4.2. Renal Tubular Epithelial Granulin Is Implicated in TLR9/IFN-α-Mediated Tubulointerstitial Injury in LN

Castellano et al. confirmed type 1 interferon is up-regulated in the RTECs. Its expression is correlated to the expansion of SLE-tubulointerstitial inflammation [67]. In this reaction, the downstream signaling molecule of toll-like receptor 9 (TLR9) is IFN-α [68]. Papadimitraki et al. reported TLR9 expression in renal tubulointerstitium in LN [69]. Granulin (GRN), a glycosylated protein, regulates cell growth, tissue development/remodeling and inflammation and also serves as an essential co-factor for TLR activation [70,71,72,73]. Huang et al. demonstrated that GRN expression in RTEC was associated with SLE-tubulointerstitial inflammation [74]. This expression can activate the TLR9–IFN-α pathway in RTEC that leads to microenvironmental inflammation in LN.

### 4.3. Urinary Excretion of β2-Microglubulin (β-2M) in SLE-Tubulointerstitial Inflammation

β-2M is a small protein constituting light chain of major histocompatibility complex (MHC) class I molecule that is normally filtered through glomeruli and reabsorbed/catabolized in proximal renal tubular cells [75]. Increased urinary excretion of β-2M has long been a biomarker for tubulointerstitial lesions [76].

### 4.4. Tamm-Horsfall Protein (THP) in SLE-Tubulointerstitial Inflammation

THP is a 7 × 10^4^ kDa macromolecule synthesized exclusively by epithelial cells in the thick ascending limb of Henle’s loop and early distal convoluted tubule, which is excreted directly into urine [77,78]. Therefore, THP can be a specific biomarker for distal renal tubules. The existence of THP in serum is believed to result from basolateral epithelial cell leakage in the thick ascending limb. Accordingly, serum THP is closely associated with fluctuation in kidney function. Consequently, it may be a biomarker across a broad spectrum of kidney disorders.

We demonstrated that lower THP excretion in patients with active LN-tubulointerstitial inflammation and increased urine excretion of β-2M, IL-6 and IL-8 reflected inflammatory activity in lupus GN and TIN [79].

Bedair et al. similarly reported decreased urinary THP as a biomarker for SLE-tubulointerstitial inflammation but not an SLE disease activity index statistically [80]. Thielemans et al. [81] discovered that serum THP was closely associated with kidney function/histological severity. They suggested its potential as a biomarker reflecting disease severity across a broad spectrum of kidney damages [81]. David et al. proposed that a low serum Tamm-Horsfall glycoprotein (STHG)/estimated (e)GFR index be a potential biomarker for renal disease activity in SLE [82].

### 4.5. Tissue and Cell Collagens: Biomarkers Associated with Kidney Fibrosis in SLE-Tubulointerstitial Inflammation

During fibrosis progression, the balance between collagen formation and degradation tilts toward its increased formation, deposition and even cross-linking but with a low-grade degradation and sear resolution. Although several soluble biomarkers for kidney damage and disease activity have been suggested for tubulointerstitial inflammation [83], there is still no reliable parameter in this regard in real life clinical practice. By using proteomic analysis, Wei et al. identified extracellular matrix (ECM) protein molecules as biomarkers for LN [84]. Based on their findings, Genovese et al. further evaluated the potential usefulness of interstitial collagen type III (PRO-C3) and type VI (PRO-C6) formation and that of collagen type III (C3M) degradation in serum and urine in SLE with/without LN [85]. Their association with histologic markers of IFTA, inflammatory cell infiltration and chronicity in these patients was concomitantly assessed. The authors discovered that the interstitial collagen turnover in PRO-C6 (in serum) and C3M (in urine) significantly correlated with histologic biomarkers of interstitial fibrosis, tubular atrophy and monocyte infiltration.

### 4.6. Fibrocyte

Fibrocytes are small population of mesenchymal cells in peripheral blood derived from bone marrow progenitors. They express features of monocytes/macrophages and also exhibit CD45, CD34, extracellular matrix proteins, type 1 collagen and α-smooth muscle actin [86,87]. More uniquely, fibrocytes play an important role in the process of fibrogenesis via the production of ECM proteins that are closely related to fibrosis and chronic inflammation [88]. Kim et al. reported that spindle-shaped coll^+^α-SMA^+^CD34^+^CD45^+^ fibrocytes were present abundantly in the peripheral blood of LN patients with interstitial fibrosis and renal dysfunction [89].

### 4.7. Ferroptosis

Ferroptosis is an iron-dependent form of cell death characterized by lipid peroxidation occurring mainly in sites of tubular segments of lupus kidney with increased acetyl-CoA synthetase long-chain family member 4 (ACSL4) serving as a pro-ferroptotic enzyme [90,91]. Alli et al. reported that intra-renal ferroptosis may represent a tissue pathological event contributing to tubular injury in human and murine LN [92].

### 4.8. CD44 Molecule

CD44 is a transmembrane glycoprotein widely expressed on surface of leukocytes (monocytes, lymphocytes, and neutrophils) and non-leukocyte cells (epithelial and fibroblastic cells). CD44 is a receptor for hyaluronan and plays various important biological/immunological roles in cell migration, proliferation, cell-matrix interaction, and presentation of TGF-β1 to its cognate receptors. These engagement reactions can induce downstream tissue fibrosis [93,94]. CD44 is only weakly expressed in the renal mesangium [95]. However, its expression is markedly enhanced in the experimental kidney injury and human GN correlating to glomerular and tubulointerstitial damages [96,97]. Wong et al. investigated the roles of CD44 in kidney inflammation and fibrosis in murine LN as well as the clinicopathological association of serum CD44 in patients with biopsy-proven class III/IV ± V LN [98]. The authors clearly proved that CD44 plays a pathogenic role in renal parenchymal inflammation and fibrosis in active LN.

### 4.9. Parvovirus B19

As mentioned above in Section 2.2.4., PVB-19 capsid antigen or protein had limitedly detection in tubular epithelial cells with infiltrating CD8-positive cells in an 11-year-old girl with spherocytosis. Although this may be rare, the detection of PVB-19-related antigens or viral particles may be a feasible way to confirm the diagnosis because it is frequently associated with SLE [50,52].

The spectrum of specific and nonspecific biomarkers for SLE-tubulointerstitial inflammation are listed in Table 1.

## 5. Controversies Regarding the Tubulointerstitial Inflammation, Tubular Atrophy and Fibrosis as Predictors for Renal Outcome in LN

The 2004 pathological classification of LN by International Society of Nephrology/Renal Pathology Society (ISN/RPS) was solely based on glomerular damage in the kidney [99]. However, glomerular damage only is not sufficient to reflect the long-term outcome of LN [100,101]. Other pathologic changes in renal biopsies of LN may comprise a spectrum of glomerular, vascular and tubulointerstitial lesions. Among these pathologic lesions, tubulointerstitial inflammation is common in LN [102]. Alsuwaida and colleagues reported that persistent tubulointerstitial inflammation was associated with poor renal outcome in LN [103]. Furthermore, a comprehensive assessment of inflammation in TI region should be included to provide better prognostic information. These doctrines have been confirmed by Wilson et al. and Gomes et al. [104,105]. The 2018 revised ISN/RPS classification incorporated the evaluation of activity index (AI) for tubulointerstitial inflammation and chronicity index (CI) for IFTA by a semi-quantitative scoring system to assess the significance of tubulointerstitial inflammation [106]. Lee et al. measured *bcl-2* expression in CD4^+^ and CD20^+^ lymphocytes of biopsied specimens and found expression of *bcl-2* was significantly higher in severe tubulointerstitial inflammation group than in the mild counterparts [107]. Duong et al. compared two scoring systems [108], total cortical interstitial inflammation score and NIH interstitial inflammation score, as predictors for chronic kidney disease (CKD) progression. They concluded, in contrast to the NIH interstitial inflammation classification, that accounting for TI in the entire parenchyma would more precisely identify LN patients at risk of CKD progression.

## 6. Potential Therapeutic Strategy for SLE-Tubulointerstitial Inflammation

It is estimated that approximately 40–45% of SLE patients develop LN and around 10% LN deteriorate into ESRD after 10 years despite therapies. Hsieh et al. demonstrated that TI damage correlated with renal function even more closely than glomerular damage [42]. Moderate to severe tubulointerstitial inflammation would become a poor prognostic sign in LN. Broder et al. [59] further revealed that moderate to severe tubulointerstitial damage, rather than tubulointerstitial inflammation, was a reliable predictor for progression to ESRD independent of eGFR or glomerular findings [109].

For SLE therapy, hydroxychloroquine is the first-line drug to reduce disease activity, morbidity and even mortality. If necessary, immunosuppressive agents including corticosteroid, azathioprine, cyclophosphamide and mycophenolate can be added on. In the past decade, major breakthrough therapies have been developed such as belimumab, anifrolumab and voclosporin. Anecdotal therapies with rituximab, tocilizumab or abatacept have also been reported. However, the disease course of SLE remains unpredictable and the morbidity/mortality are variable. Meanwhile, some of the contemporary or novel therapeutic strategies have been tried either in murine lupus or human LN.

### 6.1. Novel Therapeutic Strategies in Murine SLE

Proteasome acts as a multi-enzymatic protein complex indispensable for intracellular homeostasis. These homeostatic functions include degrading un- or mis-folded proteins, controlling cell cycle and regulating gene expression, and activating NF-κB/cytokine expression and subsequent stroma cell interactions. Bortezomib (BZ) as a selective inhibitor of 26 S proteasome has been effectively used to treat relapsed multiple myeloma [59]. Based on the evidence, Neubert et al. successfully applied BZ for protection of NZB/W F1 mice from nephritis by eliminating both short- and long-lived plasma cells through activation of terminal unfolded protein response (UPR) [110]. Subsequently, they confirmed that BZ can specifically protect podocyte ultrastructure. This important effect would successively contribute to renal protection by preserving glomerular and tubulointerstitial architectures as reported by Hainz [111].

### 6.2. Bcl-2 as a Therapeutic Target in Human Tubulointerstitial Inflammation

Different from lupus GN caused by circulating IC deposition, human LN-tubulointerstitial inflammation is characterized by in situ antigen-driven B cell clonal expansion. As described above, there is enhanced *bcl-2* expression in CD4^+^ T and CD20^+^ B cell infiltrates in the SLE-TI region [108]. Ko et al. demonstrated that *bcl-2* is also highly expressed in the TI of NZB/WF_1_ mice [112]. Furthermore, the treatment of F1 mice with selective oral inhibitor of *bcl-2* (ABT-199) could prevent proteinuria and tubulointerstitial inflammation development and prolong their survival. These results identify *bcl-2* as a potential therapeutic target for LN-tubulointerstitial inflammation.

### 6.3. Anti-Oncostatin M Antibody (Anti-OSM) Inhibits TI Lesion in Murine LN-Tubulointerstitial Inflammation

Oncostatin is a pleiotropic cytokine in IL-6 superfamily produced by activated T cells and mononuclear cells. It is a useful biomarker for lupus disease activity [113,114]. Nightingale et al. and Pollack et al. discovered human renal proximal tubular epithelial cells undergo epithelial–mesenchymal trans-differentiation (EMT) in response to OSM [115,116]. This activity suggests that OSM processes’ potential pro-fibrotic activity. Liu et al. further showed that anti-OSM antibody significantly improved EMT, inflammation and TI fibrosis in a murine LN model [117]. In addition, phosphorylated signal transducer and activator of transcription 3 (p-STAT3) rather than p-STAT1 was suppressed after anti-OSM antibody injection, which may be linked to tubulointerstitial inflammation in LN.

### 6.4. STAT3 Inhibitors Ameliorate TI Lesion in Autoimmune Lupus Mice

Individual specific STAT3 inhibitors have been tried to treat LN in MRL-*lpr/lpr* mice. By using S3I-201 (a well-known STAT3 inhibitor), stattic (a small molecule STAT3 inhibitor) or silencing of STAT3 gene and FLLL32 (a diketone analog of curcumin specific inhibitor of JAK2/STATs), respectively, Du et al., Yoshida et al. and Zu et al. found that this group of specific STAT3 inhibitors or gene knockout could ameliorate tubulointerstitial inflammation in LN [118,119,120]. Besides LN and LN-tubulointerstitial inflammation, other diseases such as diabetic nephropathy, acute kidney injury, polycystic kidney disease, and renal cell carcinoma, can implicate STAT overexpression in different renal tissues. STAT3-targeting therapy for these similar disorders can be anticipated [121].

### 6.5. Roles and Therapeutic Potential of NLRP3 Inflammasome in LN-Tubulointerstitial Inflammation

Recently, the important roles of innate immune system in the pathogenesis of SLE has been emphasized. Nucleic acids represent major self-antigens in SLE which induce autoantibodies against DNA and RNA to further form ICs. These ICs can act as damage-associated molecular patterns (DAMPs) and subsequently activate inflammasome, NLRP3, via TLRs, finally facilitating release of IL-1β, IL-18 and caspase-1 from pyroptotic innate immune cells [122,123,124]. In addition, neutrophil extracellular trap (NET) formation by SLE-neutrophils acts as an effective activator of the NLRP3 inflammasome [125]. Lorenz et al. demonstrated that NLRP3 inflammation was involved in kidney inflammation and fibrosis [126]. Oliveira et al. comprehensively reviewed the role of NLRPs inflammasomes in LN [127].

MCC950 is a highly specific inhibitor of NLRP3 inflammasome that can reduce the production of caspase-1 and proinflammatory cytokines IL-1β and IL-18. Coll et al. showed that it does not impair the production of pro-caspase 1 and pro-IL-1β by monocytes/macrophages [128]. However, it significantly suppresses the release of caspase-1 and IL-1β. Accordingly, MCC950 may primarily inhibit the assembly of the NLRP3 inflammasome rather than interfere with its activation/inhibition process [129]. The therapeutic potential of MCC950 in SLE should be explored in further research and clinical applications in tubulointerstitial inflammation/LN.

### 6.6. Macrophages as a Potential Therapeutic Target in LN-Tubulointerstitial Inflammation

Orme and Mohan reported that kidney biopsies from LN patients showed a variety of macrophage subpopulations in different compartments of the kidney [130]. Infiltration of these tissue-resident macrophages (TrMacs) may lead to the development of a hybrid proinflammatory and anti-inflammatory functional phenotypes. These mixed phenotypes were found relevant to continuous damage induced by ICs and circulating inflammatory mediators. In murine SLE, glomerular macrophages appear in the early disease course, whereas the tubulointerstitial infiltration of macrophages would become more abundant at later nephritis stage [131]. After treatment with monocytes/macrophages inhibitor, which is a macrophage polarization regulator (pathogen-associated molecular pattern activator, Pam3CSK4, abbreviated as PAM3) eliciting transformation of M2-like macrophage from M1, Horuluoglu et al. successfully improved the renal outcome in LN patients [132]. Thus, targeting macrophages is proposed as a potential new therapeutic strategy in LN [133]. Furthermore, investigations have demonstrated that macrophage polarization is controlled by multiple metabolic pathways including glycolysis, pentose phosphate pathway, fatty acid oxidation, sphingolipid metabolism, tricarboxylic acid cycle and arginine metabolism. This metabolic reprograming can be achieved by substances such as fish oil, taurine, fumaric acid, polyenylphosphatidylcholine (PPC) and some medication, including metformin and salbutamol, as reviewed by Zhao et al. [134].

These novel therapeutic agents and strategies are listed in Table 2.

## 7. Conclusions and Perspectives

**Figure 4:**
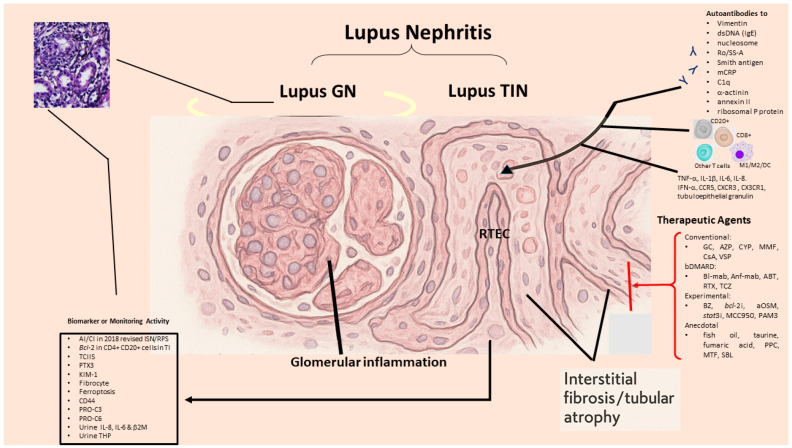
The pathogenesis, roles of various cellular and humoral factors, potential biomarkers as well as feasible therapeutic strategies for tubulointerstitial nephritis (TIN) in SLE. AI: activity index, CI: chronicity index, ISN/RPS: International Society of Nephrology/Renal Pathology Society, TI: tubulointerstitium, TCIIS: total cortical interstitial inflammation score, PTX3: pentraxin 3, KIM-1: kidney injury molecule 1, CD: cluster of differentiation, PRO-C3: interstitial collagen type III; PRO-C6: interstitial collagen type VI, mCRP: monomeric CRP, IL: interleukin, β2M: beta-2-microglobulin, THP: Tamm-Horsfall protein, C1q: complement component 1 q fragment, TNF: tumor necrosis factor, IFN: interferon, CCR: C-C chemokine receptor, CXCR: C-X-C chemokine receptor, CX3CR: C-X-X-X-C chemokine receptor, GC: glucocorticoids, AZP: azathioprine, CYP: cyclophosphamide, MMF: mycophenolate mofetil, CsA: cyclosporine A, VSP: voclosporin, bDMARD: biological disease modifying anti-rheumatic drug, Bl-mab: belimumab, Anf-mab: anifrolumab, ABT: abatacept, RTX: rituximab, TCZ: tocilizumab, BZ: bortezomib, bcl-2i: inhibitor for B cell lymphoma-2 gene encoded protein (or venetoclax), aOSM: anti-oncostatin M antibody, Stat3i: signal transducer and activator of transcription 3 inhibitor, MCC950: NLRP3 inflammasome inhibitor, PAM3: macrophage polarization regulator, PPC: polyenylphosphatidylcholine, MTF: metformin, SBL: salbutamol, GN: glomerulonephritis, RTEC: renal tubular epithelial cell.

As shown in Figure 4, differently from lupus GN that is mediated mainly by autoantibodies or preformed IC deposition with complement activation, the pathology and pathogenetic factors for SLE-tubulointerstitial inflammation are more intricate. It may implicate IC deposition, innate inflammatory process and cytotoxic T cell immune response, as well as kidney resident cell-mediated tubulointerstitial inflammation/atrophy. A presence of TIN may indicate poor prognosis for LN because of its liability to evolve into ESRD. Despite classical and more recent immunosuppressant/target therapies, the outcome of LN does not seem much improved. Accordingly, a number of novel therapeutic strategies, including proteasome inhibitors, anti-OSM (IL-6), STAT3 inhibitors, NLRP3 inflammasome inhibitors and M2 macrophage polarizing agents, have been tried in murine model with promising results. We propose that the following four prospective targets may be set to overcome clinical dilemma in SLE-tubulointerstitial inflammation:

1. Investigation of more reliable urinary biomarkers in relation to SLE-tubulointerstitial inflammation histopathology.
2. Identification of driving genes in SLE-tubulointerstitial inflammation based on bioinformatics and AI.
3. Identification of novel therapeutic modalities to transform phenotypic M1 to M2 macrophages in SLE-tubulointerstitial inflammation by hampering oxidative stresses.
4. Development of molecules to target non-coding RNAs involved in LN and SLE-tubulointerstitial inflammation.

## Figures and Tables

**Table 1:** Feasible biomarkers for SLE-tubulointerstitial inflammation.

Increased pentraxin 3 in blood and urine [66] Increased granulin expression in renal tubular epithelial cells (RTEC) [74] Increased urinary β2M [76] Decreased urinary Tamm-Horsfall protein↓ [79] Increased interstitial collagen type III (PRO-C3) and type VI (PRO-C6) in tissues [85] Spindle-shaped Coll^+^α-SMA^+^CD34^+^CD45^+^ fibrocytes in peripheral blood [89] Increased CD44 molecule expression in experimental lupus animal model [96,97] Parvovirus B-19 capsid protein in tubular epithelium [52]

**Table 2:** The novel therapeutic agents in the treatment of murine TIN.

Agents	Actions and Effectiveness
Proteosome inhibitor bortezomib (BZ) [111]	* Activating the terminal unfolding protein response for protection of podocyte ultrastructure in NZB/W F_1_ mouse
Bcl-2 inhibitor (ABT-199, venetoclax) [112]	*^,§^ Prolonging survival and prevention of proteinuria and tubulointerstitial inflammation development in NZB/W F_1_ mouse
Anti-oncostatin M antibody [117]	* Suppressing inflammation, epithelial–mesenchymal trans-differentiation (EMT) and tubulointerstitial fibrosis in murine LN model
Specific STAT3 inhibitors [118,119,120]	* Ameliorating tubulointerstitial inflammation in MRL/*lpr* mouse model
Specific NLRP3 inflammasome inhibitor MCC950 [128]	* Suppressing the release of caspase-1 and IL-1β inhibition on assembling NLRP3 inflammasome in animal SLE model
Macrophage polarization regulator (PAM3) [132,134]	Polarizing the tissue-resident macrophages into M_2_-like macrophages

TIN: tubulointerstitial nephritis; IL: interleukin; NZB/W F_1_: The 1st generation offspring from mating of New Zealand black mouse and white mouse parents; STAT: signal transducer and activator of transcription; NLRP: nucleotide-binding oligomerization domain, Leucine rich Repeat and Pyrin domain containing; Bcl-2: regulator protein, encoded by B cell lymphoma-2; * animal studies; ^§^ human studies.

## Data Availability

No new data were created or analyzed in this study. Data sharing is not applicable to this article.
